# Utility of a clinical risk scale to predict the requirement of advanced airway management in patients with a diagnosis of deep neck abscess^☆^^☆☆^

**DOI:** 10.1016/j.bjorl.2023.101360

**Published:** 2023-11-17

**Authors:** Juan Carlos Méndez Gutiérrez, Luis García-Covarrubias, Arturo Reding-Bernal, Héctor A. Velázquez Chong, Diana F. Fernández Ángel, Aldo García Covarrubias, Juan Carlos Hernández-Rivera

**Affiliations:** a Hospital de Especialidades Dr. Bernardo Sepúlveda Gutiérrez, Centro Médico Nacional Siglo XXI, Instituto Mexicano del Seguro Social, Head and Neck Department, Mexico City, Mexico; b Hospital de Especialidades Dr. Bernardo Sepúlveda Gutiérrez, CMN XXI, Instituto Mexicano del Seguro Social, Gastro-Surgery Department, Mexico City, Mexico; c Hospital General de México “Dr. Eduardo Liceaga”, Surgery Department, Mexico City, Mexico; d Hospital General De México “Dr. Eduardo Licega”, Research Department, Mexico City, Mexico; e Hospital de Especialidades Dr. Bernardo Sepúlveda Gutiérrez, CMN Siglo XXI, Instituto Mexicano del Seguro Social, Medical Research Unit on Nephrological Diseases, Mexico City, Mexico

**Keywords:** Abscess, Neck, Scale, Intubation, Risk

## Abstract

**Objectives:**

To analyze the clinical utility of a clinical risk scale to predict the need for advanced airway management in patients with deep neck abscess.

**Methods:**

Observational, analytical, cross-sectional study. Patients over 18 years old, both genders, with surgical management of a deep neck abscess, between January 1st, 2015 to December 31th, 2021, who were applied the clinical risk scale (https://7-414-5-19.shinyapps.io/ClinicalRiskScore/). The sensitivity, specificity, and predictive values of the scale were calculated based on the identified clinical outcomes. A *p* < 0.05 was considered significant.

**Results:**

A sample of 213 patients was obtained, 121 (56.8%) men, of whom 50 (23.5%) required advanced airway management. Dyspnea was the variable with the most statistical weight in our study, (*p* = 0.001) as well as the multiple spaces involvement, (*p* = 0.001) the presence of air corpuscles, (*p* = 0.001) compromise of the retropharyngeal space (*p* = 0.001) and age greater than 55 years (*p* = 0.001). Taking these data into account, were found for the clinical risk scale a sensitivity of 97% and a specificity of 65% (*p* = 0.001, 95% CI 0.856–0.984).

**Conclusions:**

The clinical risk scale developed to predict advanced airway management in patients with a diagnosis of deep neck abscess may be applicable in our environment with high sensitivity and specificity.

**Level of evidence:**

IV.

## Highlights

• Deep neck abscesses are one of the most dangerous emergencies in otolaryngology.
• The principal complication in deep neck abscesses is airway obstruction.
• It is difficult to predict which patients will require Advance Airway Management.
• Some factors have been used to create scales to stratify the risk of complications.
• The benefit of a risk scale is to prevent requirement of advanced airway management.

## Introduction

Deep neck abscesses (DNA) are one of the most frequent surgical emergencies in the otolaryngology and head and neck surgery.^1^, ^2^ The urgency in their treatment lies in avoiding complications and then reducing the morbidity and mortality derived from this.^3^, ^4^ His principal complication is airway obstruction.^4^, ^5^ In these cases, patients should receive Advance Airway Management (AAM) in addition to drain the abscess; however, it is difficult to predict which patients will require such management and which will not.^6^, ^7^, ^8^, ^9^ In recent years, some factors have been identified in order to create scales or scores to stratify the risk of complications of DNA, some with excellent results, and others have been shown to be able to predict whose will require AAM.^10^, ^11^, ^12^, ^13^

## Objective

To validate the clinical usefulness of a clinical risk scale to predict the requirement of AAM in adult patients with a diagnosis of DNA.

## Methods

### Type of study

Observational, retrospective, analytical and cross-sectional study.

### Inclusion criteria

Patients older than 18 years of both genders who have been admitted to our hospital with a diagnosis of DNA and undergoing surgery in the period from January 1, 2015 to December 31, 2021.

### Exclusion criteria

Patients who presented any of the following circumstances: pregnant, postpartum, or lactating patients; advanced airway management (orotracheal intubation or tracheostomy) already established in another hospital; with uncomplicated nodal peritonsillar abscesses; history of chemotherapy or radiation therapy; patients in whom the deep neck abscess was associated with malignancy, cervical trauma, or previous surgical site infections; or who had not accepted the proposed treatment.

### Elimination criteria

Patients with incomplete clinical records, or with missing two or more study variables.

### Methodology

The clinical records of the included patients in the described period of time were reviewed. Airway management prediction was performed according to the CLINICAL RISK SCALE (CRS) using the calculator located on the website: https://7-414-519.shinyapps.io/ClinicalRiskScore/.

### Sample size calculation

It was calculated at 18 patients per group based on a sensitivity of the clinical risk scale >80%, reported in the article by Lin et al.^14^

### Type of test

Generic 1-tailed binomial test, Proportion p2 = 0.8, Probability of error *α* = 0.05, Power (probability of error 1−β) = 0.80, Proportion p1 = 0.5.

### Statistical analysis

Descriptive statistics were used for the clinical and sociodemographic characteristics collected. To determine the normality of the distribution, a Kolmogorov–Smirnov test was performed. For quantitative variables with normal distribution, the Student’s *t*-test was used and for those with non-normal distribution, the Mann Whitney *U* test was used. For qualitative variables, Pearson’s Chi-Square test, or Fisher’s E. test was used as appropriate, according to the absence or presence of the analyzed clinical outcome. (Advanced airway management) for a ROC (Receiver Operating Characteristic) curve, the area under the curve and a cut-off point for the CRS were estimated using the logistic regression model proposed by Lin et al.^14^ With this new cut-off point, sensitivity, specificity, positive predictive value, negative predictive value, overall concordance, as well as the Kappa index were estimated. All *p-*values were considered at a significance level <0.05. Statistical analyzes were performed using the statistical software STATA® version 15.1. (StataCorp®, 201, United States).

### Ethical aspects

This study does not represent any risk as it is retrospective, observational, analytical, and cross-sectional. In the same way, no research product will expose the identity of the participating individuals and they data were only used for the purposes of this research. Likewise, the researchers declare that they have no conflicts of interest with the results of the study. The protocol was approved by the local ethics and research committee with approval number: R-2022-3601-046.

## Results

A sample of 213 patients was obtained, 121 (56.8%) men, of whom 50 (23.5%) required AAM. The mean age was 52 ± 16 years. Orotracheal intubation was used in 40 (80%) of the patients who required AAM and in the other 10 (20%) tracheostomy was used. (Table 1) The most frequent location of the collection was the suprahyoid region in 139 (65.3%) patients, of which 41 (29.4%) required AAM. Multiple space involvement was observed in 77% and was present in 98% of those requiring AAM (*p* = 0.001). All those who required AAM had compromise of the retropharyngeal space (*p* = 0.001). In a bivariate manner, (intubated or not) significant differences were found in the presence of dyspnea, multiple space involvement, presence of affected space corpuscles, and platelet/lymphocyte ratio (Table 1).

**Table 1:** Demographic and clinical data according to whether the patients were intubated or not.

Variable	Total	Mechanic ventilation		*p*-Value
	(n = 213)	Yes (163)	No (50)	
Gender				
Male, n (%)	121	23 (53.5)	98 (57.6)	
Female, n (%)	92	20 (46.5)	72 (42.3)	0.749^a^
Dyspnea				
Yes, n (%)	80	42 (97.6)	38 (22.3)	
No, n (%)	133	1 (2.3)	132 (77.6)	<0.001^b^
Multiple space				
Yes, n (%)	164	42 (97.6)	122 (71.7)	
No, n (%)	49	1 (2.3)	48 (28.2)	<0.001^b^
Air corpuscles				
Yes, n (%)	95	30 (69.8)	65 (38.2)	
No, n (%)	118	13 (30.8)	105 (61.8)	<0.001^a^
Space				
Infrahyoid, n (%)	65	0 (0.0)	65 (0.3)	
Supra hyoid, n (%)	139	35 (0.8)	104 (0.6)	
Retropharynx, n (%)	9	8 (0.2)	1 (0.0)	<0.001^b^
Age, media (ED)	52.3	56.1 (16.0)	51.4 (16.0)	0.087^c^
Neutrophils, media (ED)	0.8	0.8 (0.04)	0.82 (0.04)	0.592^d^
Plaquettes/lymphocytes index, mean (DE)	259.6	277.4 (59.6)	255.8 (59.6)	<0.001^d^
Albumin, mean (ED)	3.7	3.8 (0.4)	3.7 (0.4)	0.1388^d^
Score test, mean (ED)	0.2	0.69	0.16	<0.001^d^

SD, standard deviation (*p*-value significant ≤0.05).

a Pearson’s Chi-Square test.

b Fisher’s E. test.

c Student’s *t*-test.

d Mann–Whitney *U* test.

**Figure 1:**
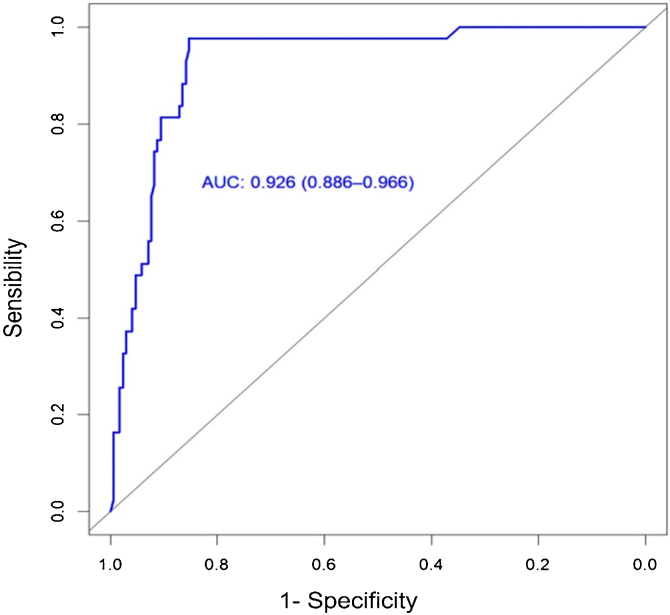
ROC (Receiver Operating Characteristic) Curve. The estimated Area Under the Curve (AUC) was 0.926 with a cut-off point of 0.451. With this new cut-off point, a sensitivity of 97.7% and a specificity of 85.3% was obtained.

Using an ROC curve, the area under the estimated curve was 0.926 with a cut-off point of 0.451. With this new cut-off point, a sensitivity of 97.7% was obtained, and a specificity of 85.3% (Fig. 1). A sensitivity of 0.975 and a specificity of 0.659 (*p* = 0.001, 95% CI 0.856–0.984) were found for the clinical risk scale. The median score obtained on the clinical scale for the group of patients who required AAM was 70.6% risk, while in the group of those who did not require AAM the median score was 4.2% risk, managing to increase specificity and the predictive value + with the new cut-off point proposed (Table 2).

**Table 2:** Diagnostic indicators using the new cut-off point of 0.451 compared to the cut-off point of 0.226 of Lin et al.^14^

Variable	Cut point	
	0.451	0.226
Sensitivity	97.67%	97.67%
Specificity	85.29%	77.64%
Predictive value +	62.68%	52.50%
Predictive value −	99.31%	99.24%
Overall value	87.79%	81.69%
Kappa	0.68	0.57

## Discussion

A clinical scale was applied in patients with a diagnosis of deep neck abscess, to predict the risk of requiring AAM and thus validate its usefulness in our population.^14^ We found a predominance in the male gender, however, this was not significant (*p* = 0.383). Regarding age, after 55 years the risk of requiring advanced airway management increases (*p* = 0.05) similar to that reported in other studies, where the risk was higher after 60 years.^14^ Dyspnea was the most relevant clinical data, present in 2% of those who did not require AAM, against 98% of those who did, (*p* = 0.001) consistent with other publications, where it was reported to occur in 6.6% of patients who do not require AAM (*p* < 0.001).^15^

The formation of gas in infectious processes is associated with the presence of anaerobic bacteria, contributing to its spread, and is therefore considered a prognostic factor for airway management.^15^ Air corpuscles were present in 72% of those who required AAM, while only 28% of those did not (*p* = 0.001). Other investigators have observed air corpuscles in up to 71.7% of those requiring AAM.^15^ The percentage of neutrophils in the blood and the platelet/lymphocyte ratio were found to be elevated in both groups (*p* = 0.595 and 0.396 respectively). In some publications, lower values of these parameters have been found in those who did not require AAM,^16^, ^17^ probably because the patients sought care in advanced stages of the disease, when both biomarkers were already very elevated, except in those with more severe infection, who presented increased ranges, making it possible to identify those with a higher risk of requiring AAM.^17^, ^18^, ^19^, ^20^

In the context of an infectious disease, hypoalbuminemia is an indicator of metabolic stress, therefore, in DNA, it can serve as a marker of severity and prognosis of AAM.^21^, ^22^, ^23^ This variable was not significant (*p* = 0.138) possibly because the patients had a very deteriorated metabolic state and significant comorbidities, unlike other authors who have reported hypoalbuminemia as a significant variable (*p* ≤ 0.001) due to the better general conditions of their patients.^23^, ^24^, ^25^ One of the strengths of this study is that it was carried out in a single center, therefore, the reliability of the results is high, since all the patients were treated under similar conditions and following the same diagnostic-therapeutic protocol, in addition to being a reference hospital, the sample obtained was quite significant (213 patients in 7 years). However, as retrospective study, there may be selection bias as most patients are in more advanced stages of the disease.

The main benefit of this clinical risk scale is its implementation as a tool that will allow the timely identification of patients who may require advanced airway management, with a positive impact on their prognosis by reducing the mortality rate when performing intubation or tracheostomy. timely manner.

## Conclusion

The clinical risk scale developed by Lin et al. in patients diagnosed with deep neck abscess may be applicable in the Mexican population.

This clinical risk scale can be implemented as an additional tool that, together with the knowledge and experience of medical personnel, will allow us to identify those patients more safely with a higher risk of requiring advanced airway management. Detecting these patients in a timely manner has a positive impact on their prognosis since the mortality rate can be reduced by carry out important actions such as notifying support staff (anesthesiology, nursing, inhalation therapy, etc.) and prepare all the equipment necessary to intubate or perform a tracheostomy.

## Funding

None.

## Conflicts of interest

The authors declare no conflicts of interest.
